# Bilateral plantar deep cleft

**DOI:** 10.11604/pamj.2017.28.312.14502

**Published:** 2017-12-15

**Authors:** Caio Cavalcante Machado, Fred Bernardes Filho

**Affiliations:** 1 Reumatology Division, Department of Medical Clinics, Ribeirao Preto Medical School, University of São Paulo, Ribeirão Preto, Brazil; 2 Dermatology Division, Department of Medical Clinics, Ribeirão Preto Medical School, University of São Paulo, Ribeirão Preto, Brazil

**Keywords:** Foot diseases, toe joint, foot bones

## Image in medicine

A 37-year-old previously healthy woman presented with fever, headache, erythematous rash and joint pain. She did not experience cough, coryza, sore throat, diarrhea, nausea, or vomiting. Positive findings on physical examination included erythematous follicular macules and papules on the trunk and arms, swollen ankles and hyperemic sclera. We found a coincidental clinical finding: bilateral plantar deep cleft Radiography of the feet showed third and fourth metatarsal shortening along without osteodystrophic features. Laboratory tests revealed positivity for immunoglobulin M antibodies to Zika vírus. Blood count, electrolytes, serum measurements of calcium, phosphorus and parathyroid hormone were normal. The diagnosis of Zika virus infection was made and fortuitously, brachymetatarsia was identified. Brachymetatarsia is an uncommon condition of the foot characterized by shortening of the metatarsal of the foot and when present, it is usually asymptomatic. It can be either uni or bilateral and can affect any of the metatarsal bones. Brachymetatarsia may be congenital or idiopathic in etiology and may be associated with systemic diseases such as pseudo-hyperparathyroidism, Turner's syndrome, Down's syndrome, Apert syndrome, enchondromatosis, multiple epiphyseal dysplasia, sickle cell anemia and poliomyelitis. Brachymetatarsia may affect one or more metatarsals and may be unilateral or bilateral.

**Figure 1:**
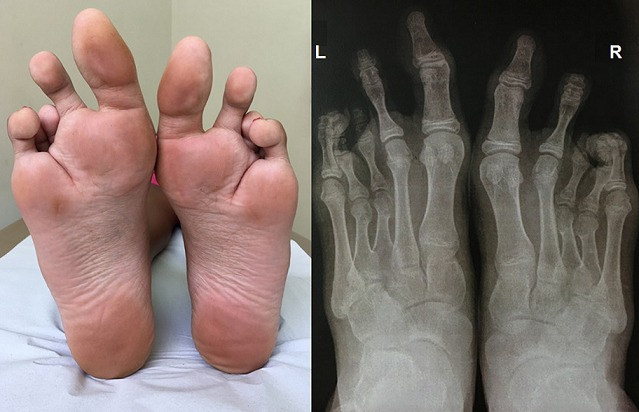
Plantar deep cleft and radiography of the feet showing third and fourth metatarsal shortening along without osteodystrophic features

