# Corrigendum: Bilateral plantar deep cleft

**DOI:** 10.11604/pamj.2018.30.59.16068

**Published:** 2018-05-28

**Authors:** Fred Bernardes Filho

**Affiliations:** 1 Dermatology Division, Department of Medical Clinics, Ribeirão Preto Medical School, University of São Paulo, Ribeirão Preto, Brazil

**Keywords:** Corrigendum, Foot diseases, toe joint, foot bones

## Abstract

This corrigendum corrects article “Bilateral plantar deep cleft” (The Pan African Medical Journal. 2017;28:312. doi:10.11604/pamj.2017.28.312.14502).

## Corrigendum

The original version of this article [1] had the author’s name wrongly abbreviated on PubMed as Filho FB instead of Bernardes Filho F.
